# Investigating xylose metabolism in recombinant *Saccharomyces cerevisiae* via ^13^C metabolic flux analysis

**DOI:** 10.1186/1475-2859-12-114

**Published:** 2013-11-18

**Authors:** Xueyang Feng, Huimin Zhao

**Affiliations:** 1 Department of Chemical and Biomolecular Engineering, Institute for Genomic Biology, Urbana, USA; 2 Departments of Chemistry, Biochemistry, and Bioengineering, University of Illinois at Urbana-Champaign, Urbana, IL 61801, USA

**Keywords:** ^13^C-MFA, Fungal xylose pathway, Maintenance, Cofactor engineering

## Abstract

**Background:**

To engineer *Saccharomyces cerevisiae* for efficient xylose utilization, a fungal pathway consisting of xylose reductase, xylitol dehydrogenase, and xylulose kinase is often introduced to the host strain. Despite extensive *in vitro* studies on the xylose pathway, the intracellular metabolism rewiring in response to the heterologous xylose pathway remains largely unknown. In this study, we applied ^13^C metabolic flux analysis and stoichiometric modeling to systemically investigate the flux distributions in a series of xylose utilizing *S. cerevisiae* strains.

**Results:**

As revealed by ^13^C metabolic flux analysis, the oxidative pentose phosphate pathway was actively used for producing NADPH required by the fungal xylose pathway during xylose utilization of recombinant *S. cerevisiae* strains. The TCA cycle activity was found to be tightly correlated with the requirements of maintenance energy and biomass yield. Based on *in silico* simulations of metabolic fluxes, reducing the cell maintenance energy was found crucial to achieve the optimal xylose-based ethanol production. The stoichiometric modeling also suggested that both the cofactor-imbalanced and cofactor-balanced pathways could lead to optimal ethanol production, by flexibly adjusting the metabolic fluxes in futile cycle. However, compared to the cofactor-imbalanced pathway, the cofactor-balanced xylose pathway can lead to optimal ethanol production in a wider range of fermentation conditions.

**Conclusions:**

By applying ^13^C-MFA and *in silico* flux balance analysis to a series of recombinant xylose-utilizing *S. cerevisiae* strains, this work brings new knowledge about xylose utilization in two aspects. First, the interplays between the fungal xylose pathway and the native host metabolism were uncovered. Specifically, we found that the high cell maintenance energy was one of the key factors involved in xylose utilization. Potential strategies to reduce the cell maintenance energy, such as adding exogenous nutrients and evolutionary adaptation, were suggested based on the *in vivo* and *in silico* flux analysis in this study. In addition, the impacts of cofactor balance issues on xylose utilization were systemically investigated. The futile pathways were identified as the key factor to adapt to different degrees of cofactor imbalances and suggested as the targets for further engineering to tackle cofactor-balance issues.

## Introduction

Biofuel, especially cellulosic biofuel, plays an increasingly important role in sustainable energy supply and greenhouse gas emissions reduction [1]. Thanks to a number of breakthroughs in metabolic engineering and synthetic biology, a series of industrial microorganisms such as *Escherichia coli*[2] and *Saccharomyces cerevisiae*[3,4] have been engineered to use non-food feedstock to produce a variety of biofuels. Among all these endeavors, one of the most promising strategies is to engineer *S. cerevisiae* to utilize xylose for bioethanol production. To this end, a heterologous xylose pathway identified from fungal species such as *Scheffersomyces stipitis* and *Candida tenuis* is often introduced into *S. cerevisiae* by functionally expressing xylose reductase (XR), xylitol dehydrogenase (XDH), and xylulose kinase (XKS). When engineering the fungal xylose pathway in recombinant *S. cerevisiae* strains, the unbalanced utilization of various cofactors by XR and XDH [5] is often believed to be an issue that may affect the performance of xylose fermentation. Basically, NADPH is preferred to be consumed by XR with the production of NADP^+^, while NAD^+^ is required by XDH. Such cofactor imbalance accounts for xylitol accumulation due to the insufficient regeneration of NAD^+^ for the XDH reaction from the heterologous xylose pathway and the endogenous central carbon metabolism, which leads to low ethanol yield [4,6,7].

Numerous efforts have been made in the past three decades to alter the cofactor specificity of XR and XDH. For example, protein engineering was used to increase the specificity of XR to NADH [8-10], and improve the specificity of XDH to NADP^+^[11]. In the meantime, a series of metabolic engineering strategies were used to optimize the cofactor utilizations in xylose metabolism. In general, three independent approaches have been developed, including: 1) altering the preference of XR to use NADH as the cofactor [12-17]; 2) altering the preference of XDH to use NADP^+^ as the cofactor [10,18-21]; and 3) engineering cofactor dependent metabolic pathways in the native *S. cerevisiae* such as 6-phosphogluconate dehydrogenase in the oxidative pentose phosphate pathway [22] and glyceraldehyde-3-phosphate dehydrogenase in the glycolysis pathway [23]. As expected, the decreased xylitol production and enhanced ethanol yield could be achieved by using any of these strategies, which raises an interesting question: which strategy or strategies should be pursued to push the xylose utilization to the theoretical limit?

To address this question, detailed understanding of the interplays between the heterologous xylose pathway and the native host metabolism is required. In the past decade, the *in vivo* metabolic behaviors of recombinant *S. cerevisiae* strains when metabolizing xylose have been analyzed by stoichiometric flux analysis [24,25] and metabolomics analysis [26,27]. In this study, we attempted to rigorously investigate the global effect of the heterologous xylose pathways by selecting six representative engineered xylose utilizing *S. cerevisiae* strains from our previous studies [28] and applying ^13^C-metabolic flux analysis (^13^C-MFA) to systemically characterize the *in vivo* carbon flux distributions. We found that the oxidative pentose phosphate pathway was actively used in the recombinant *S. cerevisiae* strains in order to supply enough NADPH for the fungal xylose pathway. Carbon fluxes into the TCA cycle were regulated in response to the metabolic burdens of expressing a heterologous xylose pathway in nutrient limited medium. Based on the discovery from ^13^C-MFA, an *in silico* study was carried out to evaluate the impact of cofactor engineering strategies and cell maintenance energy on xylose-based ethanol production under different fermentation conditions. To augment previous efforts on engineering *S. cerevisiae* for efficient xylose utilization, this study provides quantitative readouts of xylose metabolism and uncovers new insights about metabolic regulations of a heterologous xylose pathway.

## Material and methods

### Strains, media, and culture conditions

The *S. cerevisiae* strains used in this study are summarized in Table 1. All *S. cerevisiae* strains were stored in 25% glycerol at −80°C. To culture *S. cerevisiae* strains, seed cultures were grown in YPAD media (1% yeast extract, 2% peptone, 0.01% adenine hemisulfate, 20 g/L glucose) at 30°C for overnight. The seed cultures were then inoculated (0.1%, v/v) into the defined minimal medium as described elsewhere [29,30], with 5 g/L xylose as the sole carbon source. The yeast strains were grown with triplicates at 30°C, 250 rpm in capped culture tubes (14 mL) containing 6 mL defined minimal medium to achieve the oxygen limited condition. Cell growth was monitored at OD_600_. In the exponential phase, two samples were taken for growth rate calculation and extracellular metabolites measurement.

**Table 1:** ***S. cerevisiae *** **strains used in this study**

***S. cerevisiae *** **strains**	**Origins of XR**	**Origins of XDH**	**Origins of XKS**
CF1	*Aspergillus flavus*	*Candida dubliniensis*	*Nectria haematococca*
CF3	*Aspergillus nidulans*	*Aspergillus niger*	*Pencillium chrysogenum*
CF9	*Aspergillus flavus*	*Pichia guilliermondii*	*Candida dubliniensis*
CS1	*Candida dubliensis*	*Nectria haematococca*	*Aspergillus niger*
CS5	*Zygosaccharomyces rouxii*	*Aspergillus niger*	*Nectria haematococca*
CS10	*Candida dubliensis*	*Scheffersomyces stipitis*	*Podospora anserina*

XR: xylose reductase; XDH: xylitol dehydrogenase; XKS: xylulose kinase.

For ^13^C tracer experiments in defined minimal medium, the same culture conditions were used to culture *S. cerevisiae* strains, except that the carbon source was replaced with 5 g/L [1-^13^C] xylose (Cambridge Isotope Laboratories). For ^13^C tracer experiments in YPAX medium (1% yeast extract, 2% peptone, 0.01% adenine hemisulfate, 40 g/L xylose), 0.1% inoculum was transferred from the seed culture to YPAX medium with 40 g/L [1-^13^C] xylose as the carbon source. For all ^13^C tracer experiments, *S. cerevisiae* strains were grown in duplicates, and the cells (i.e. biomass) were harvested at the mid-log phase based on the growth curve previously determined using non-labeled xylose in defined minimal medium and YPAX medium [28].

The concentrations of extracellular metabolites, including xylose, xylitol, and ethanol were analyzed by HPLC equipped with a refractive index detector (Shimadzu Scientific Instruments, Columbia, MD) and an HPX-87H column (BioRad, Hercules, CA). The flow rate was 0.6 mL/min at 60°C using 5 mM sulfuric acid as the mobile phase. No byproducts but xylose was detected in the supernatant.

### Isotopic analysis

For GC-MS measurement of isotopomer labeling patterns in proteinogenic amino acids, the biomass was harvested by centrifugation and hydrolyzed using 6 M HCl (24 h at 100°C). The amino acids were derivatized in 50 μl of tetrahydrofuran and 50 μl of *N*-(tert-butyl dimethylsilyl)-*N*-methyl-trifluoroacetamide (Sigma-Aldrich). A gas chromatograph (GC2010, Shimadzu) equipped with a DB5-MS column (J&W Scientific, Folsom, CA) and a mass spectrometer (QP2010, Shimadzu) were used for analyzing metabolite labeling profiles. Three types of charged fragments were detected by GC-MS for Ala, Gly, Ser, Asp, Glu, Phe, and Leu: the [M-57]^+^ group (containing unfragmented amino acids); and the [M-159]^+^ or [M-85]^+^ group (containing amino acids that had lost an α-carboxyl group). For each type of fragments, the labeling patterns were represented by M0, M1, M2, etc., which were fractions of unlabeled, singly labeled, and doubly labeled amino acids (Additional file 1: Text S1). The effects of natural isotopes on isotopomer labeling patterns were corrected by previously reported algorithms [31]. The ^13^C enrichment of proteinogenic amino acids was calculated as ${}^{13}C\%=\frac{\sum_{i=1}^{C}\left(i\cdot M_{i}\right)}{C}$, where *M*_*i*_ is the GC-MS isotopomer fraction for a given amino acid; *C* is the total number of carbon atoms in the amino acid molecule.

### ^13^C metabolic flux analysis

The central metabolic pathway map of recombinant *S. cerevisiae* strains was generated based on genome annotation from the KEGG database (Kyoto Encyclopedia of Genes and Genomes) and previous fluxomics studies [24,25,29,32]. The simplified pathway map includes the fungal xylose pathway, oxidative and reductive pentose phosphate pathway, glycolysis, futile pathways, transport pathways between cytosol and mitochondria, and TCA cycle (Additional file 1: Text S2). As discussed before [33], to develop a pseudo-steady-state flux model, the xylose uptake rates were measured at the mid-log phase and normalized to 100 units. The biomass composition of *S. cerevisiae* strains was referred to a previous study [29], and used as loose constraints for the fluxes into building block synthesis (i.e., allowed to have variations of up to 20%). To determine the remaining unknown fluxes of the metabolic network, a nonlinear optimization problem was formulated to iteratively search a set of fluxes that can satisfy the reaction stoichiometry and minimize the objective function [33]: $\epsilon \left(v_{n}\right)={\sum_{i=1}^{a}\left(\frac{M_{i}-N_{i}\left(v_{n}\right)}{\delta_{i}}\right)}^{2}$, where *v*_*n*_ are the unknown fluxes to be optimized in the program, *M*_*i*_ are the measured isotopomer labeling patterns of proteinogenic amino acids, *N*_*i*_ are the model-simulated isotopomer labeling patterns of proteinogenic amino acids using isotopomer mapping matrices (IMM), *δ*_*i*_ are the standard deviations of the GC-MS data from two biological replicates. The nonlinear optimization was finished by using “fmincon” command in MATLAB (MathWorks, USA). The optimization was run for 100 times with different initial guesses to search for a likely global solution for the unknown fluxes. The observed and simulated isotopomer labeling patterns of proteinogenic amino acids were compared in Additional file 1: Figure S1. The confidence intervals of the calculated fluxes were generated via a Monte Carlo approach as described in our previous research [33]. In short, the isotopomer labeling patterns of the proteinogenic amino acids were randomly perturbed for 100 times within the standard derivation. For each perturbed dataset, the ^13^C-MFA was applied and the flux distributions were calculated. Then, the standard derivations of each flux were derived from such 100 simulations.

Since the relative activities of isoenzymes cannot be decided, the cofactor balancing for NADPH was not included in the mass balancing of ^13^C-MFA. For the NADH production, only the glycolysis pathway and the TCA cycle (without considering isocitrate dehydroganase) was taken into calculation. Considering that no fermentation products (e.g., ethanol) can be detected, it was hence assumed that all of the NADH was used for ATP production via oxidative phosphorylation.

### *In silico* simulations

A stoichiometric model developed by Christen and Sauer [29] was modified and used in this study to simulate the ideal ethanol production under different xylose utilization conditions. Such stoichiometric model includes the same metabolic pathways as those in the ^13^C-MFA. A parameter “*f*” was introduced to describe the degree of cofactor imbalance in the heterologous xylose pathway: Xylose + *f* ∙NADPH - *f* ∙NADH + ATP → Xylulose 5-phosphate + *f* ∙NADP^+^ - *f* ∙NAD^+^ + ADP. To simplify the model simulation, the cofactors for aldehyde dehydrogenase (EC 1.2.1.5), malic enzyme (EC 1.1.1.38), and the isocitrate dehydrogenase (EC 1.1.1.42) were set as NADPH. Similar to the ^13^C-MFA, NADH was only considered to be produced from the glycolysis pathway and the TCA cycle and was consumed for either ATP production through oxidative phosphorylation or ethanol production through the fermentation pathway.

To find the optimal ethanol production, we normalized the xylose uptake rate into 100 units and sampled multiple biomass yields, growth associated maintenance requirements, and oxygen uptake conditions as the model inputs. For each of the model input, the metabolic fluxes were constrained by reaction stoichiometry [34] to find the maximal ethanol yield (equation 1). The mathematical optimization was then solved by linear optimization solver “linprog” in MATLAB (MathWorks, USA):

(1) $$\begin{array}{l} \max v_{\mathrm{EtOH}}\\ s.t.\;S\left(f,\mathrm{GAM}\right)\cdot v=0\\ \;\mathrm{lb}\leq v\leq \mathrm{ub}\\ \;v_{\mathrm{Xylose}}=100\\ \;v_{\mathrm{Biomass}}\;\mathrm{are}\;\mathrm{fixed}\\ \;v_{\mathrm{Oxygen}}\in \left[0,ub_{O2}\right]\;\\ \;f\in \left[0,1\right] \end{array}$$

where *S* is the stoichiometric matrix and is correlated with the degree of cofactor imbalance in the xylose pathway (i.e., *f*) and the growth associated maintenance requirement (i.e., GAM in mmol ATP/g DCW), *v* is the vector of metabolic fluxes, *v*_*Xylose*_ is the xylose uptake rate normalized as 100 units, *v*_*Oxygen*_ is the normalized oxygen uptake flux, *v*_*Biomass*_ is the normalized biomass yield, *lb* and *ub* are the lower and upper boundary of metabolic fluxes, respectively, *ub*_*O2*_ is the upper boundary of the oxygen uptake level set in the model.

### Enzymatic activity assay

The enzyme activities of XR and XDH were analyzed in a 96-well plate using a Biotek Synergy2 microplate reader (Winooski, VT) by monitoring the absorbance at 340 nm for depletion or generation of NAD(P)H as described previously [28]. The specific activities were reported as units (i.e. micromoles of NAD(P)H reduced or oxidized per minute) per milligram of protein. Cultures in the defined minimal medium or YPAX medium were harvested at mid-log phase. Cell mass equivalent to an OD of 20 was collected for lysis with YPER Extraction Reagent (Pierce, Rockford, IL). BCA Protein Assay Reagent (Pierce) was used to determine the protein concentrations and Albumin Standard (Pierce) was used for the protein concentration standard curve. The XR reaction mixture was made as 50 mM KH_2_PO_4_ buffer (pH 7), 0.2 mM NADPH or 0.2 mM NADH, and 200 mM xylose. The XDH reaction mixture was made 50 mM KH_2_PO_4_ buffer (pH 7), 50 mM MgCl_2_, 1 mM NAD^+^, and 200 mM xylitol.

In order to measure the specific activity of XKS, a modified procedure with a glycerol kit manufactured by R-Biopharm (Darmstadt, Germany) was used. Since the XKS assay was coupled to pyruvate kinase and lactate dehydrogenase [35], the NADH depletion can be monitored and linearly related to XKS activity. The standard protocol of the glycerol kit was followed, with the substitution of galactose kinase with crude enzyme XKS and using 5 mM *D*-xylulose rather than glycerol as the substrate [28].

## Results

### Metabolic behaviors of selected *S. cerevisiae* strains

To investigate the effects of heterologous xylose pathways on native carbon metabolism of *S. cerevisiae*, six recombinant strains with different origins of XR, XDH and XKS in the xylose pathway (Table 1) were selected from our previous study [28]. In that previous study, the XR, XDH and XKS enzymes cloned from different microorganisms were assembled via a high-throughput method (i.e. DNA assembler [36]) to construct a library of xylose pathways. The resulting library of xylose pathways was then transformed into *S. cerevisiae*, from which some of the recombinant strains were successfully selected based on their significantly improved metabolic behaviors in terms of growth rates, xylose consumption rates, and ethanol yields when culturing in YPAX medium.

Among the six strains, three of them (i.e. CF1, CF3, and CF9) were originally identified as the “fast-growers” with higher growth rates (ranging from 0.10 ~ 0.14 h^-1^, Table 2) and ethanol yields (ranging from 0.18 ~ 0.25 g/g) during library screening in YPAX medium, while the other three strains (i.e. CS1, CS5, and CS10) were found to be “slow-growers” with lower growth rates (ranging from 0.05 ~ 0.07 h^-1^) and ethanol yields (ranging from to 0.07 ~ 0.15 g/g). When growing the selected strains in minimal medium with xylose as the sole carbon source under oxygen limited conditions, the production of ethanol and other products (e.g. acetate, glycerol, and xylitol) was not detected, indicating the consumed xylose was almost exclusively used for biomass production. Such fermentation behaviors were also reported by a recent research on evolutionary engineering of *S. cerevisiae* for efficient xylose utilization [37]. Among the selected strains in this study, diverse metabolic behaviors were identified, with growth rates ranging from 0.02 ~ 0.05 h^-1^, xylose uptake rates ranging from 0.54 ~ 3.08 mmol/g DCW/h, and biomass yield ranging from 0.08 ~ 0.47 g/g. The “fast-growers” and “slow-growers” were hence re-grouped based on the performances in the minimal medium. In general, when culturing in minimal medium, one of the “fast-growers” (i.e. CF3) had lower growth rates than two of the “slow-growers” (i.e. CS1 and CS10) identified in YPAX culturing. The diverged fermentation profiles of the same recombinant strains in the minimal medium and the YPAX medium suggested that the metabolic responses to the xylose utilization were different under diverse culturing conditions.

**Table 2:** **Fermentation profiles of *S. cerevisiae *** **strains**

***S. cerevisiae *** **strains**	**Minimal medium**			**YPAX medium**			
	**Xylose uptake (mmol/g/h)**	**Growth rate (h**^**-1**^**)**	**Biomass yield (g/g)**	**Xylose uptake (mmol/g/h)**	**Growth rate (h**^**-1**^**)**	**Biomass yield (g/g)**	**Ethanol yield (g/g)**
CF1	0.69 ± 0.21	0.046 ± 0.005	0.469 ± 0.149	2.07 ± 0.21	0.14 ± 0.01	0.45 ± 0.02	0.25 ± 0.03
CF3	0.83 ± 0.30	0.026 ± 0.002	0.184 ± 0.064	1.83 ± 0.09	0.13 ± 0.01	0.47 ± 0.02	0.20 ± 0.03
CF9	1.07 ± 0.17	0.048 ± 0.004	0.299 ± 0.024	1.44 ± 0.07	0.10 ± 0.01	0.46 ± 0.02	0.18 ± 0.01
CS1	3.08 ± 0.21	0.034 ± 0.009	0.078 ± 0.033	1.40 ± 0.28	0.05 ± 0.00	0.24 ± 0.01	0.07 ± 0.00
CS5	0.57 ± 0.03	0.018 ± 0.003	0.234 ± 0.032	1.42 ± 0.28	0.06 ± 0.00	0.28 ± 0.01	0.11 ± 0.02
CS10	0.54 ± 0.14	0.030 ± 0.011	0.369 ± 0.125	1.43 ± 0.29	0.07 ± 0.00	0.33 ± 0.02	0.15 ± 0.01

The One-step ANOVA for the biomass yields in minimal medium returns *p* value as low as 0.0102, concluding that there is strong evidence that the biomass yields in the all the groups (i.e., CF1, CF3, CF9, CS1, CS5, and CS10) differ. The fermentation profiles of *S. cerevisiae* strains in YPAX medium were referred to previous report [28].

### Carbon metabolism of recombinant *S. cerevisiae* strains

**Figure 1:**
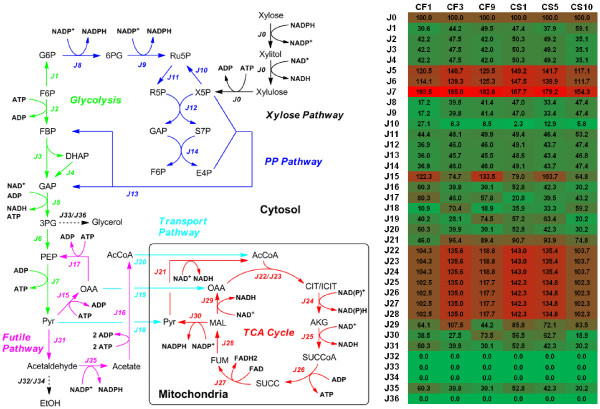
**Heatmap of metabolic flux distributions of *S. cerevisiae *strains.** The xylose uptake flux in each of the *S. cerevisiae* strains was normalized as 100 units.

By feeding [1-^13^C] xylose into the minimal medium and analyzing the corresponding isotopomer enrichment patterns in proteinogenic amino acids, the carbon fluxes through the central metabolic networks were calculated for the selected *S. cerevisiae* strains (Figure 1). The xylose uptake fluxes of each recombinant *S. cerevisiae* strain were normalized to relative flux value as 100. Based on ^13^C-MFA, in all of the *S. cerevisiae* strains, the oxidative pentose phosphate pathway had high fluxes (relative flux value as 17.2 ~ 47.4), which is consistent with transcriptional analysis showing the up-regulation of the oxidative pentose phosphate pathway in xylose growth [8]. The metabolic fluxes split at the node of F6P to enter either oxidative pentose phosphate pathway or the glycolysis pathway, at the ratio close to 1:1. The flux into reductive pentose phosphate pathway merged with the glycolytic flux at the node of GAP with a ratio close to 1:1. The futile cycle such as pyruvate carboxylase and phosphoenolpyruvate carboxykinase was found to be active and served as the major pathway for net production of oxaloacetate to refuel the TCA cycle in mitochondria.

**Figure 2:**
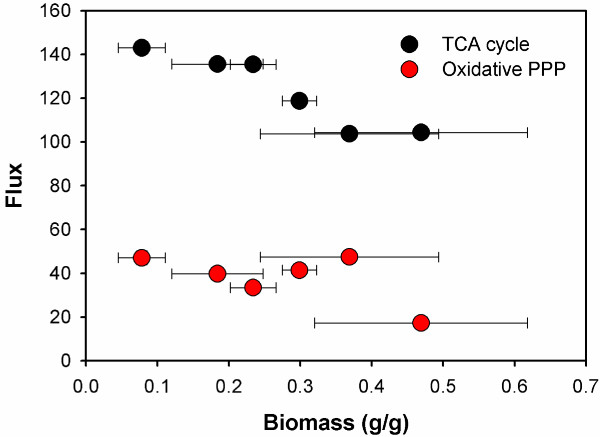
Correlation between biomass yields (g DCW/g xylose) and the fluxes in TCA cycle or oxidative pentose phosphate pathway.

While the similar flux distribution patterns were followed by all the selected strains, differences among various xylose metabolisms were still observed. Particularly, the metabolic fluxes through the TCA cycle differed from strain to strain (ranging from 102.3 ~ 142.3 for relative flux value), and were found to be inversely correlated with the biomass yields (Figure 2). For example, the CF1 strain had nearly the lowest TCA cycle activity (relative flux value as 102.5) but the highest biomass yield (0.469 ± 0.149 g/g), while the CS1 strain had the highest TCA cycle activity (relative flux value as 142.3) but the lowest biomass yield (0.078 ± 0.033 g/g). Since two carbons were lost as CO_2_ in one TCA cycle, the strong activity of TCA cycle will make poor use of the carbon substrates, which may explain the inverse correlations between biomass yield and TCA cycle activity. The fluxes into oxidative pentose phosphate pathway could be correlated with biomass yields in two ways. On one hand, the NADPH produced from the oxidative pentose phosphate pathway were the major source for synthesis of biomass building blocks. On the other hand, the CO_2_ released from the oxidative pentose phosphate pathway can lead to the decrease of biomass yields. The trade-off between such two effects could explain that neither positive or negative correlation was observed between fluxes into oxidative pentose phosphate pathway and biomass yields (Figure 2).

### Energy metabolism of recombinant *S. cerevisiae* strains

The balancing of cofactors including NADPH, NADH and ATP was not included when calculating the carbon flux by ^13^C-MFA, due to the lack of information about the relative activities of isoenzymes. For instance, the isoenzymes of isocitrate dehydrogenase can use either NAD^+^ (EC 1.1.1.41) or NADP^+^ (EC 1.1.1.42) in the TCA cycle. Therefore, it becomes difficult to quantify exactly how much NADH or NADPH was produced by isocitrate dehydrogenase without knowing the relative activities of different isoenzymes. On the other hand, it has been suggested in many ^13^C-MFA studies [38,39] that the flux distributions can be calculated independently without the cofactor balancing, followed by using the carbon flux distributions to uncover the *in vivo* utilization of cofactors. In this study, the active oxidative pentose phosphate pathway contributes to 11 ~ 74% of NADPH required by the fungal xylose pathway and the building block synthesis in biomass production. The aldehyde dehydrogenase (EC 1.2.1.5), malic enzyme (EC 1.1.1.38), and the isocitrate dehydrogenase (EC 1.1.1.42) may also contribute to the NADPH production in xylose metabolism.

**Figure 3:**
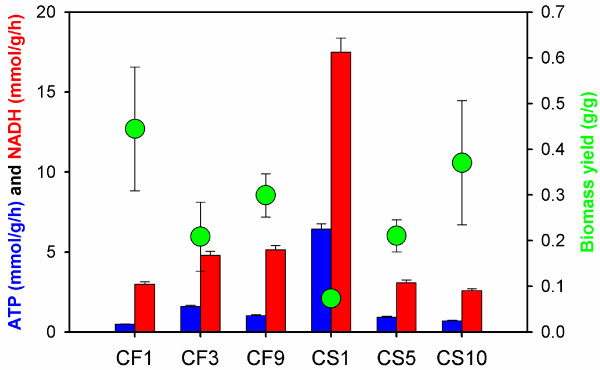
Correlation between biomass yields (g DCW/g xylose) and the net produced ATP and NADH as required for cell maintenance (mmol ATP/g DCW).

For NADH and ATP metabolism, the TCA cycle in recombinant *S. cerevisiae* strains showed strong activities, generating huge amount of NADH as the redox for oxidative phosphorylation. As a result, significant amount of ATP was produced, which leads to high requirements for maintenance energy in xylose metabolism of the recombinant *S. cerevisiae* strains (Figure 3). Interestingly, since the TCA cycle was mainly used for energetic instead of biosynthetic purpose, the requirements for cell maintenance energy were found to be inversely correlated with the biomass yields. That is, the higher TCA cycle activity is, the more ATP was generated through oxidative phosphorylation as maintenance energy, while more carbons were lost as CO_2_, resulting in lower biomass yields.

### *In silico* simulation of xylose metabolism in recombinant *S. cerevisiae* strains

**Figure 4:**
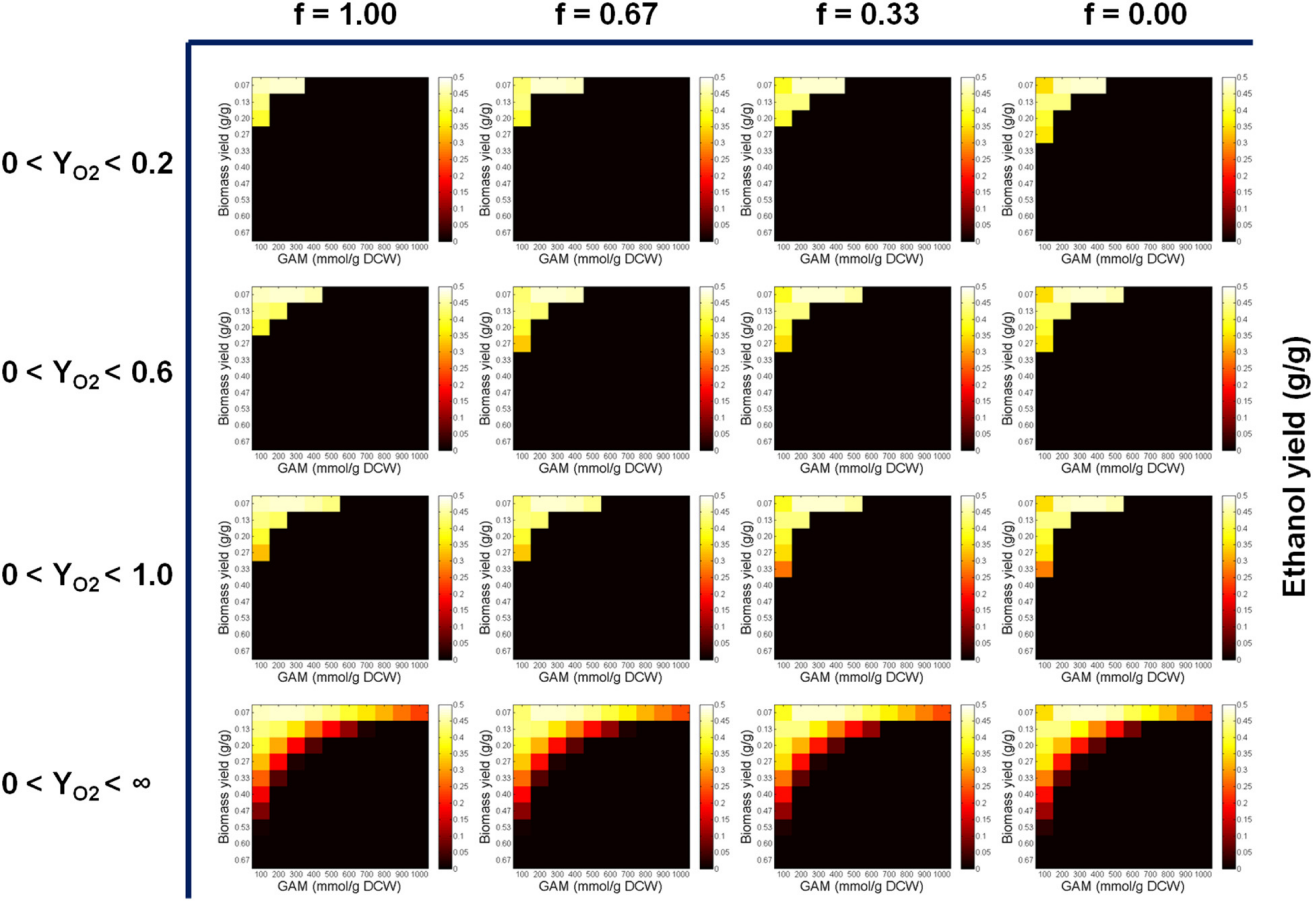
***In silico *simulations of maximal ethanol yield (g ethanol/g xylose) by using different cofactor engineering strategies (i.e., “*f*” as in the pathway of Xylose +** ***f *·NADPH - *f *·NADH + ATP → Xylulose 5-phosphate +** ***f *·NADP**^**+ **^**- *f *·NAD**^**+ **^**+ ADP) under various fermentation conditions with combinations of diverse biomass yields, GAM, and aerobic conditions (represented by Y**_**O2 **_**as the oxygen uptake in mol O**_**2**_**/mol xylose).**

To evaluate the impact of cofactor imbalance issue on xylose metabolism of recombinant *S. cerevisiae* strains, we applied a stoichiometric model for *in silico* simulation of the maximal ethanol yields (Figure 4). Since the XR enzymes are not strictly NADH or NADPH dependent, using the different XR enzymes in the heterologous xylose pathway could lead to various degrees of cofactor imbalance in *S. cerevisiae* strains. In general, we introduced “*f*” (ranging from 0 to 1) as a parameter to quantify the degree of cofactor imbalance in the heterologous xylose pathway (i.e., Xylose + *f* ∙NADPH - *f* ∙NADH + ATP **→** Xylulose 5-phosphate + *f* ∙NADP^+^ - *f* ∙NAD^+^ + ADP). For each value of “*f*”, we sampled different biomass yields, growth associated maintenance requirements (i.e., GAMs), and oxygen uptake conditions as the model inputs and searched for the maximal ethanol yields under the given growth condition and with the designated xylose pathway.

**Figure 5:**
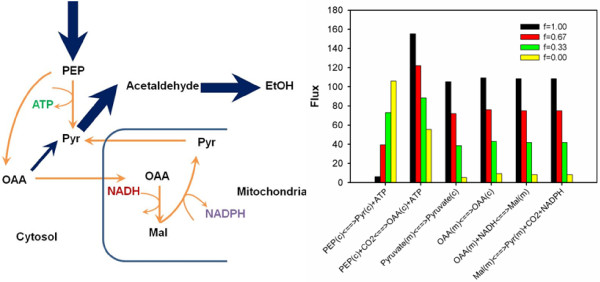
**The futile pathways flexibly used to achieve the optimal ethanol production under different cofactor engineering strategies, under aerobic conditions (i.e., 0 < Y**_**O2**_ **< ∞), with GAM set as 300 mmol ATP/g DCW.** The orange arrows indicate the futile pathways. The thickness of the blue arrows is proportional to the relative flux value.

Based on the *in silico* simulation, the ideal ethanol yield (i.e., over 95% of the theoretical yield) can be only achieved within a narrow range of biomass yields and GAMs. When biomass yield and/or GAM are high, the requirement for ATP becomes so strong that the NADH can no longer be used for ethanol production, but instead fueled into oxidative phosphorylation for ATP production. Therefore, no ethanol can be produced under these fermentation conditions. The cofactor-balanced xylose pathway (i.e., *f* = 0 as in bacterial isomerase pathway) could indeed be more beneficial to the xylose-based ethanol production than its counterparts (i.e., *f* = 0.33, 0.37, 1.00), by providing a wider range of fermentation conditions for optimal fermentation conditions. The advantages of using cofactor-balanced pathway are more significant when oxygen uptake level is lower. However, the ideal ethanol production can be possibly achieved in all of the oxygen conditions regardless of cofactor imbalance issue. The metabolic flux distributions that led to optimal ethanol production have similar patterns, including a negligible fluxes into oxidative pentose phosphate pathway, small fluxes (relative flux value less than 35) into TCA cycle, and strong fluxes towards ethanol production (relative flux value higher than 150). However, the fluxes into futile pathways were found to be very flexible corresponding to different degrees of cofactor-imbalance issues (Figure 5). For example, the fluxes of conversing oxaloacetate to malate with the consumption of one NADH were found to be positively correlated with the degrees of cofactor-imbalance issues. That is, the greater the cofactor-imbalance issue was, the more fluxes were diverted into this futile pathway to consume excessive NADH.

### ^13^C tracer experiments of *S. cerevisiae* strains in nutrient rich medium

**Figure 6:**
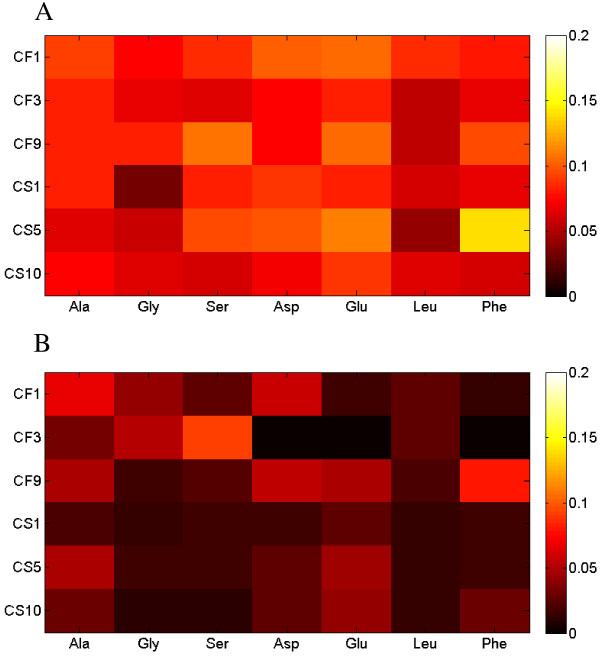
^**13**^**C enrichment of proteinogenic amino acids in *S. cerevisiae *strains cultured in A) defined minimal medium and B) YPAX medium.** Note: the mass spectrum of Asp, Glu and Phe in CF3 strain cultured in YPAX medium had too much noise to derive the reliable readouts of ^13^C enrichment.

**Figure 7:**
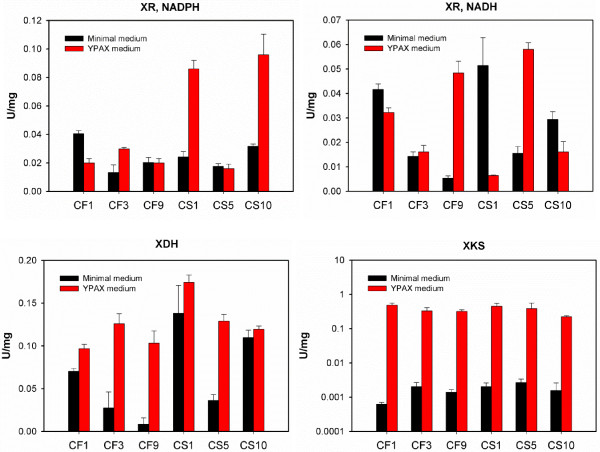
**Enzyme activities of xylose reductase (XR), xylitol dehydrogenase (XDH), and xylulose kinase (XKS) in *S. cerevisiae *strains.** The activities of XRs with both NADPH and NADH as the cofactors were measured.

In this study, we cultured strains in the oxygen limited conditions with defined minimal medium based on several considerations. First, as reported by many groups, few *S. cerevisiae* strains can grow in defined minimal medium under anaerobic conditions with xylose as the sole carbon source, since the oxygen is needed to resolve the cofactor imbalance [40]. On the other hand, the oxygen-limited condition has been suggested as optimal for ethanol production by an *in silico* simulation [4,40]. In addition, the application of ^13^C-MFA strictly requires the using of defined minimal medium for culturing recombinant *S. cerevisiae* strains. However, many labs, including ours, often choose nutrient-rich medium (e.g. YPAX) to create yeast mutants and characterize the xylose fermentation. Compared to the previous characterization of the same strains in nutrient-rich medium (i.e. YPAX), the metabolic behaviors are significantly different in defined minimal medium in terms of growth rates and ethanol production. In order to confirm that the discoveries from the ^13^C-MFA in minimal medium is also applicable to the studies of xylose utilization in rich medium, we have designed another set of isotopic tracing experiments by providing 4% [1-^13^C] xylose into the YPAX medium. The ^13^C enrichments of all the amino acids detected were only 20 ~ 50% of their counterparts from the minimal medium (Figure 6), indicating that the nutrient transport rather than *de novo* synthesis was the major pathway for building block production. As revealed by ^13^C-MFA in this study, the GAM plays a pivotal role in xylose utilization, especially in the stressful cultivation conditions. With the building blocks, such as amino acids, provided by the direct uptake from the medium instead of *de novo* synthesis, nutrient-rich medium is less stressful than the minimal medium, leading to a lower maintenance requirement and better fermentation performance. In addition, compared to those in the nutrient-rich medium, the *in vitro* enzyme activities of XR and XDH activities in the minimal medium (Figure 7) were within the same level [28]. However, the activities of XKS were about two magnitudes lower (0.001 ~ 0.003 U/mg compared to 0.1 ~ 0.4 U/mg), which could be another reason for the inconsistent metabolic behaviors of the same strain in different media.

## Discussion

In spite of thorough *in vitro* investigation of the heterologous xylose pathway, the *in vivo* metabolic behaviors of recombinant *S. cerevisiae* strains in xylose metabolism still remain largely as a black box. To make it worse, the conclusions drawn from the *in vitro* analysis may not be extended into the *in vivo* studies, considering the difference between intracellular and extracellular environments. In this case, ^13^C-MFA becomes one of the valuable tools to provide rigorous and quantitative readouts of *in vivo* network-scale metabolic behaviors. As discovered by ^13^C-MFA in this study, the requirements for maintenance energy [41] of the recombinant *S. cerevisiae* strains for xylose utilizations were so high that no ethanol can be produced. The metabolic burdens of expressing the heterologous enzymes in nutrient limited medium could account for the high requirements for maintenance energy during xylose utilization. Our previous ^13^C-MFA studies have compared the flux distributions in wild-type *S. cerevisiae* strain without expressing heterologous proteins and the engineered *S. cerevisiae* strains expressing heterologous XR, XDH, and XKS in glucose cultures [42] and found that the metabolic burdens of expressing the heterologous enzymes in nutrient limited medium led to 30 ~ 38% increase of TCA cycle fluxes in the recombinant strains so that more ATP was produced through oxidative phosphorylation to satisfy the higher requirement for the maintenance energy. The similar metabolic responses (i.e., more flux into TCA cycle) to metabolic burdens of heterologous protein expression in carbon-limited medium were also uncovered recently in several other ^13^C-MFA study of recombinant *Pichia pastoris*[43-45]. In these studies, the up-regulated TCA cycle was correlated to the decreased biomass yield since more carbons got lost as CO_2_ instead of being used for building block synthesis in TCA cycle. In this study, we found the similar linkage between the decreased biomass yield and the increased flux in TCA cycle, confirming that the metabolic burdens of expressing heterologous proteins could also play pivotal roles in xylose utilization.

It is believed that the cofactor-balanced pathway (e.g., bacterial isomerase pathway) is more advantageous for ethanol production. As suggested by the *in silico* simulation in this study, adopting the cofactor-balanced pathway does not show significant advantages to the cofactor-imbalanced pathways in terms of the maximal ethanol yield that can be achieved. However, it indeed provides a wider range of fermentation conditions for optimal ethanol production, especially under the oxygen limited conditions. Compared to the cofactor-imbalanced pathway, the cofactor-balanced pathway does not generate NADH, which can be more beneficial for cell growth when the oxygen uptake level is low, as the capacity to convert NADH to ATP via oxidative phosphorylation is limited. In addition, the futile pathways were found to be the key pathway for the optimal ethanol production in both cofactor-imbalanced and cofactor-balanced pathway, since they could be flexibly adjusted to adapt to different cofactor usages. It is hence possible that the futile pathways could be engineered in the future to tackle the cofactor imbalance issue in xylose utilization. Beside the cofactor imbalance issue, another issue that may need the attention is the metabolic burden caused by heterologous protein expression in stress conditions. As illustrated from both the *in silico* simulation and the ^13^C-MFA in this study, no ethanol can be produced when metabolic burden becomes too large to be neglected. In addition to the effects on ethanol yields, expressing the heterologous fungal pathway in *S. cerevisiae* strains also had impacts on other physiological parameters, such as growth rate and xylose consumption rate. As shown in Table 2, the growth rate of CS5 was poor while the xylose consumption rates of CS5 and CS10 were much lower than those of the other yeast strains. Deciphering the mechanisms behind the aforementioned effects could be challenging for ^13^C-MFA in this study since not only the intracellular metabolism rewiring but also the protein properties such as protein solubility, and catalytic capacities could be involved in affecting the growth rate and xylose consumption rate. Therefore, the comprehensive characterization of enzyme properties needs to be finished in the future.

In summary, by systemically applying ^13^C-MFA to a series of recombinant xylose-utilizing *S. cerevisiae* strains, the interplays between the fungal xylose pathway and the native host metabolism were uncovered. The oxidative pentose phosphate pathway of *S. cerevisiae* strains was activated to provide NADPH for the fungal xylose pathway. The TCA cycle was found to be tightly regulated in response to the requirements of maintenance energy caused by heterologous protein expression. Based on *in silico* simulations, reducing the maintenance energy can be a crucial strategy to improve ethanol production. The addition of exogenous nutrients such as amino acids to reduce the *de novo* synthesis of biomass building blocks can be an efficient strategy to reduce the maintenance energy. Also, metabolic engineering strategies, such as directed evolution, can be potentially helpful to reduce the maintenance requirement and improve the xylose based ethanol production.

## Competing interests

The authors declare that they have no competing interests.

## Authors’ contributions

XF and HZ conceived this study. XF performed the experiments; XF and HZ interpreted and analyzed data. XF and HZ wrote the manuscript and prepared a revised version. All authors approved the final version.

## Supplementary Material

Additional file 1: Text S1 Isotopomer labeling patterns of proteinogenic amino acids. The variations between the biological replicates were less than 2%. **Text S2.** Metabolic flux calculation by ^13^C-MFA. **Figure S1.** Measured and simulated isotopomer labeling patterns of proteinogenic amino acids in enzyme-based-library of *S. cerevisiae* strains. Click here for file
