# Supplementary material for: Investigating xylose metabolism in recombinant Saccharomyces cerevisiae via 13C metabolic flux analysis
Source: Microb Cell Fact. 2013 Nov 18;12:114. doi: 10.1186/1475-2859-12-114 (PMC3842631; doi:10.1186/1475-2859-12-114)

**Supporting Information**

**Supplementary Text S1: Isotopomer labeling patterns of proteinogenic amino acids. The variations between the biological replicates were less than 2%.**

**Supplementary Text S2: Metabolic flux calculation by ^13^C-MFA**

**Figure S1** Measured and simulated isotopomer labeling patterns of proteinogenic amino acids in enzyme-based-library of *S. cerevisiae* strains.


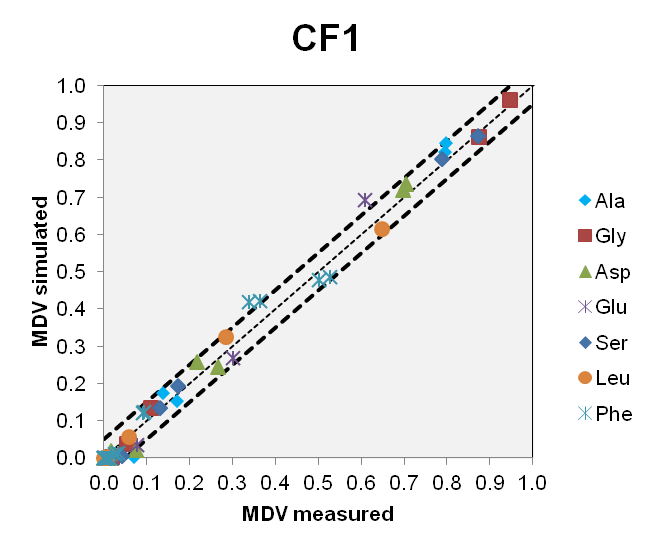

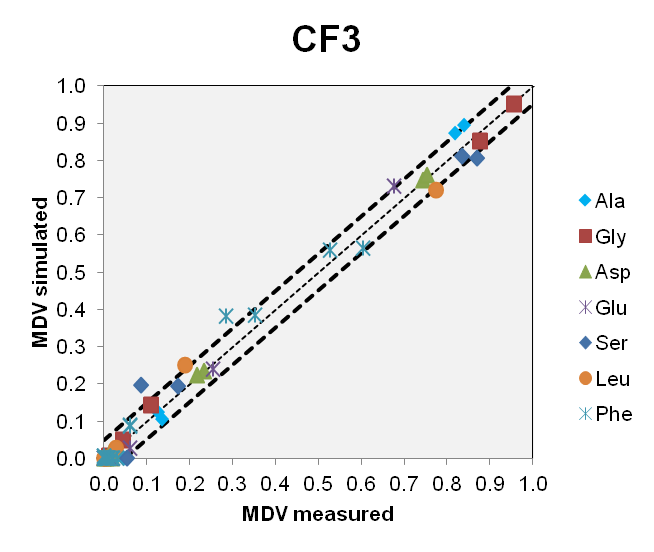

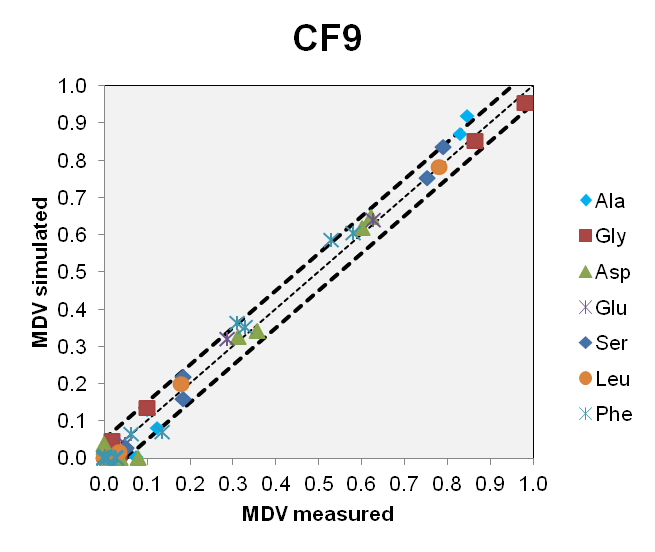

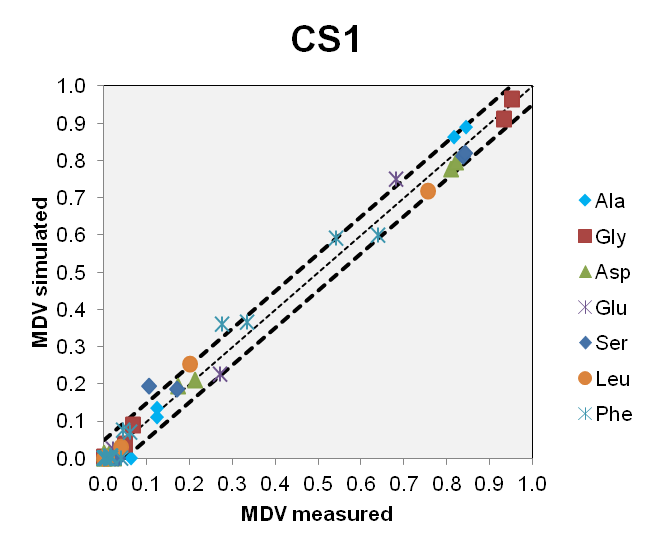

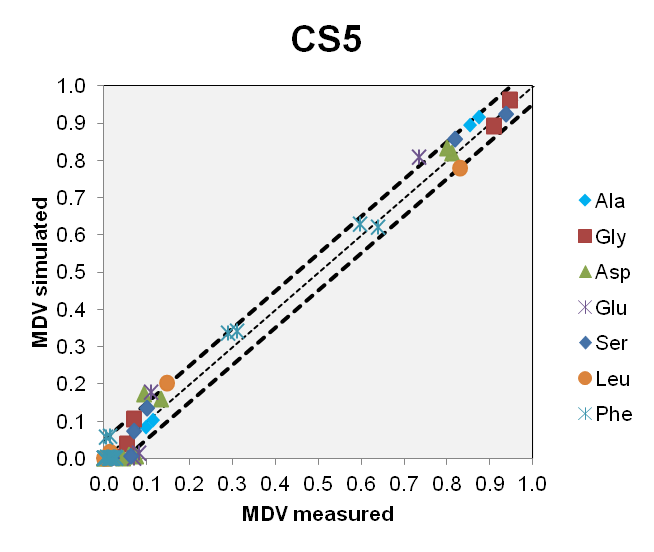

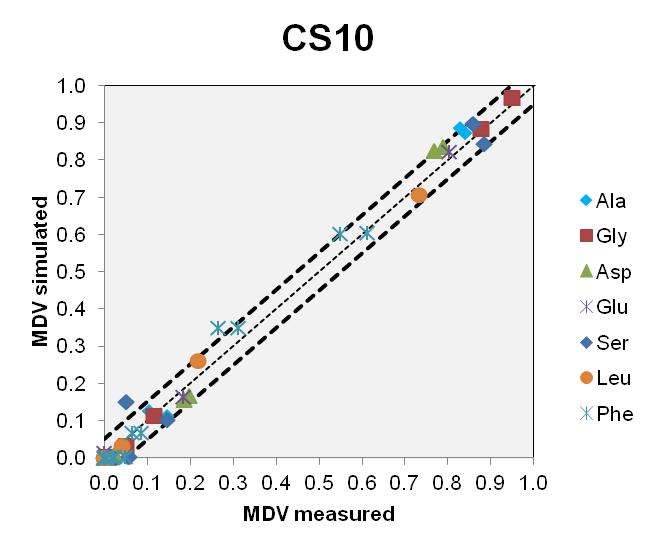

Supplement: Additional file 1: Text S1 — Isotopomer labeling patterns of proteinogenic amino acids. The variations between the biological replicates were less than 2%. Text S2. Metabolic flux calculation by 13C-MFA. Figure S1. Measured and simulated isotopomer labeling patterns of proteinogenic amino acids in enzyme-based-library of S. cerevisiae strains. [file 1475-2859-12-114-S1.docx]
